# Oxidative DNA Damage Signalling in Neural Stem Cells in Alzheimer's Disease

**DOI:** 10.1155/2019/2149812

**Published:** 2019-11-13

**Authors:** Marcelina Kieroń, Cezary Żekanowski, Anna Falk, Michalina Wężyk

**Affiliations:** ^1^Laboratory of Neurogenetics, Department of Neurodegenerative Disorders, Mossakowski Medical Research Centre Polish Academy of Sciences, 5 Pawinskiego Street, 02-106 Warsaw, Poland; ^2^Regulomics Group, Faculty of Mathematics, Informatics and Mechanics University of Warsaw, 2 Banacha Street, 02-097 Warsaw, Poland; ^3^Department of Neuroscience, Karolinska Institutet, Biomedicum D7, 171 77 Stockholm, Sweden

## Abstract

The main pathological symptoms of Alzheimer's disease (AD) are *β*-amyloid (A*β*) lesions and intracellular neurofibrillary tangles of hyperphosphorylated tau protein. Unfortunately, existing symptomatic therapies targeting A*β* and tau remain ineffective. In addition to these pathogenic factors, oxidative DNA damage is one of the major threats to newborn neurons. It is necessary to consider in detail what causes neurons to be extremely susceptible to oxidative damage, especially in the early stages of development. Accordingly, the regulation of redox status is crucial for the functioning of neural stem cells (NSCs). The redox-dependent balance, of NSC proliferation and differentiation and thus the neurogenesis process, is controlled by a series of signalling pathways. One of the most important signalling pathways activated after oxidative stress is the DNA damage response (DDR). Unfortunately, our understanding of adult neurogenesis in AD is still limited due to the research material used (animal models or post-mortem tissue), providing inconsistent data. Now, thanks to the advances in cellular reprogramming providing patient NSCs, it is possible to fill this gap, which becomes urgent in the light of the potential of their therapeutic use. Therefore, a decent review of redox signalling in NSCs under physiological and pathological conditions is required. At this moment, we attempt to integrate knowledge on the influence of oxidative stress and DDR signalling in NSCs on adult neurogenesis in Alzheimer's disease.

## 1. Introduction

Alzheimer's disease (AD) is characterized by a massive loss of neurons and synapses in the cerebral cortex. Currently, 47.5 million people worldwide are suffering from dementia, and this number is growing by 7.7 million annually (World Health Organization, WHO, 2017). WHO estimates that in 2030, this number will increase to 75.6 million people, and in 2050, it will almost triple to 135.5 million, among which AD accounts for 60-70%. One of the main pathologies of AD is related to the misprocessing of amyloid precursor protein (APP) leading to the accumulation of *β*-amyloid (A*β*) peptides. The major toxic product is 42 amino acid-long peptide (A*β*42) aggregating into extracellular plaques or forming toxic intracellular oligomers [1–3]. The second major pathological hallmark of AD is insoluble neurofibrillary tangles (NFT) aggregating in cellular bodies and dendrites of neurons. NFTs are composed of tau proteins, which are hyperphosphorylated and released from microtubules following caspase activation upon oxidative stress damage response induced by A*β* oligomers (Serrano-Pozo et al. 2011, Liu et al. 2015). So far, the existing symptomatic therapies targeting A*β* or tau remain ineffective. Since existing therapeutic approaches focus to late pathological mechanisms, the efforts to find a much earlier causal factor in AD should be increased. In this light, growing hopes lie in the research on neural stem cells (NSCs) in AD and, hence, in cellular therapies.

Both AD pathology and NSC biology remain in a tight connection with oxidative stress and DNA damage response (DDR). Despite numerous studies, the exact roots of oxidative stress in AD remain unclear. Most likely, it is the intersection of both exogenous lifestyle-related mutagenic detrimental factors and endogenous factors related to the energetic needs of neurons. At a higher concentration, A*β* induces production of reactive oxygen species (ROS) in cortical neurons through the activation of NADPH oxidase, what alters oxidative-redox balance (Shelat et al. 2008, Cheignon et al. 2017), crucial for the proliferation/differentiation cycle of NSCs [4]. A*β* also influences the mitogen-activated kinase (MAPK) and Notch signalling [5], both affecting the lifecycle of NSCs (Traiffort and Ferent 2015, Faigle and Song 2013, Kim and Wong 2015). It has been recently evidenced that A*β* impaired NSCs' viability and proliferation and indirectly blocked neurogenic differentiation, by disrupting mitochondrial signalling of self-renewing NSCs (Ribeiro et al. 2018). This study brought a new perspective to rethink the molecular targets relevant for endogenous NSC-based strategies in AD. The effects of A*β* peptides on NSCs are still not well understood and remain controversial, and the necessity for their study has been outlined recently [6]. The second major pathological hallmark of AD is an accumulation of hyperphosphorylated tau protein, induced upon oxidative stress signalling. The phosphorylation/dephosphorylation cycle of intracellular tau is indispensable for NSCs' migration capability, especially toward the sites of the injury [7]. Mutated tau in frontotemporal dementia affected the differentiation of NSCs into astrocytes, which displayed an increased vulnerability to oxidative stress and enhanced protein ubiquitination [8]. Similarly, impaired splicing of tau in radial glial-like cells (RGs) differentiated from APP-mutated embryonic stem cells was linked with affected astrocytic differentiation [9]. In general, both amyloid and tau pathologies are accompanied by an oxidative response (Liu et al. 2015), which has a direct implication to NSCs' biology.

Oxidative DDR, accompanying the A*β* and tau pathology, is manifested by recruitment of several DNA damage-sensing kinases, which can phosphorylate hundreds of proteins, including tumour suppressing and cell cycle regulating proteins (Sherman 2013). Several of them, which depends on the ataxia telangiectasia mutated (ATM) signalling, were found to promote quiescent NSC (qNSC) activation (Barazzuol et al. 2017). Conversely, neurons in vulnerable regions of AD brain display reduced signalling downstream the ATM kinase (Shen et al. 2016). Another study showed an activation of the ATM signalling and mobilization of the DDR network during the progression of AD dementia (Katsel et al. 2013). According to Katsel et al., activation of the ATM signalling is a protective mechanism occurring through the interaction with the p53 protein, which, however, weakened due to intensifying oxidative stress. Furthermore, in human neuron-like cells, the levels of ATM and other DDR components were downregulated during neuronal differentiation, with a consequent attenuation of DDR in nondividing neurons versus dividing cells (Biton et al. 2006). Overall, several experiments collectively suggest a link between DDR signalling and NSC general biology and differentiation (rev. Sherman et al. 2013).

In conclusion, the pathogenesis of AD still reveals its other side, especially in terms of potential causative factors. Oxidative stress, together with its entire signalling network is an important pathogenic process in AD, starting from the disturbances already at the stage of precursor cells of neurons. A lot of research is needed to translate this knowledge into applied medical science and thus to create early and effective therapeutic methods. In this respect, the notion of disturbance in redox-sensitive NSCs seems to meet the expectations of both primary and applied research. Therefore, in this review, we attempt to integrate the current state of knowledge on the importance of NSC biology in the course of AD, emphasizing the regulatory role of oxidative stress and DDR signalling.

## 2. Neural Stem Cells and Adult Neurogenesis

The identification of NSCs in the adult brain (Altman and Das, 1965, Erikson et al. 1998) raised the possibility that these cells may act as a source for regeneration, via either the direct repair or maintenance of brain plasticity. Active adult neurogenesis is spatially restricted under normal conditions to two specific “neurogenic” brain regions. One is the subgranular zone (SGZ) in the dentate gyrus (DG) of the hippocampus where new dentate granule cells are generated. Second is the subventricular zone (SVZ) of the lateral ventricles where neurons are born in order to migrate through the rostral migratory stream to the olfactory bulb to become interneurons [10]. Each of these zones can be modelled in vitro using pluripotent stem cells or adult NSCs (Azari and Reynolds 2016). In these neurogenic niches, there are cells of various types. In the SVZ, there are NSCs (type B cells) that extend their basal processes to blood vessels and apical processes through the ependymal cell layer to contact the cerebrospinal fluid in the ventricle [11]. Type B NSCs convert to transient amplifying progenitors (type C cells) [12], which then become neuroblasts (type A cells). Next, neuroblasts form a chain and migrate into the olfactory bulb where they migrate radially and differentiate into different subtypes of interneurons. In the SGZ, the radial glia-like NSCs (named RGLs or type 1 cells) are located between the inner granule cell layer and hilus, where they provide intermediate progenitor cells (Seri et al. 2001), which show limited proliferation cycles before they generate neuroblasts (Berg et al. 2015). Neuroblasts migrate along the SGZ and develop into immature neurons, which migrate into the granule cell layer to differentiate into dentate granule neurons [13]. Critical to the final fate of NSCs is their lifecycle behaviour, involving quiescence or activation of the cell cycle. By entering the cell cycle, NSCs choose different modes of division: asymmetric (yielding self-renewing NSCs and progenitors) or symmetric (yielding either self-renewing NSCs or non–self-renewing progenitors). Neurogenic dynamics, expressed by the ultimate fate of the cells, are restricted by the fact that progenitors may differentiate into a particular cell type or stay multipotent [14]. Different subtypes of NSCs are predefined to produce neurons, oligodendrocytes, or astrocytes, but never all three at the same time (Ortega et al. 2013). All of these “choices” of NSCs are affected by various stresses, such as the oxidative one. Specific populations of NSCs may exhibit a predisposition for certain activities, such as asymmetric division or quiescence, and this should be (re-)established in detail in AD patients. Overall, such a variety of NSCs and their state of activation in the context of the occurrence and course of AD have extensive research and therapeutic potential. Different models of the identity and activation status of NSCs have been proposed (Gonçalves et al. 2016). Such a study in the context of AD is now possible thanks to the protocols of reprogramming of patients' peripheral cells (fibroblasts, lymphocytes) to NSCs.

Furthermore, stress-related ROS production was found to promote the exit from the stem cell state and spontaneous neuronal differentiation (Hu et al. 2018). The critical issue in this regard is to define which NSC subtypes and daughter cells play a crucial role in pathological conditions in chronic oxidative stress. Next, NSCs' fate relies on various growth factors. These regulatory proteins are either secreted by NSCs themselves or by the surrounding cells, including neurotransmitters or small signalling molecules delivered via the cerebrospinal fluid from neurons and astrocytes. The factors regulating neural stem fate are, among others, vascular endothelial growth factor (VEGF), promoting NSC self-renewal; neurotrophin 3 (NT3), promoting long-term cellular wellbeing [15]; and FGF-2 (Kang and Hébert, 2015) and IGF-1, promoting proliferation of neural precursor cells (NPCs) and production of new neurons (Hsieh et al. 2004). NSCs are also influenced by the extracellular matrix (ECM) proteins such as laminin or proteoglycans [16]. Several morphogens, including bone morphogenic protein (BMP), inhibit neurogenesis and promote the formation of astroglia in the SVZ. The Notch protein, activated by the expression of *SOX2*, causes the NSCs to pass into NPCs. WNT protein and sonic hedgehog (SHH), also activated by *SOX2*, are associated with brain organogenesis but also maintenance of NPCs in the SVZ [17].

Overall, adult neurogenesis relies on the balance between proliferation and differentiation of NSCs/NPCs corresponding to states of quiescence or activation. NSC proliferation, migration, and differentiation into the defined neuronal phenotype, further integrating into the synaptic transmission, are controlled by a variety of endogenous factors, as exemplified above, and exogenous environmental triggers which can be both beneficial and detrimental. Neurogenesis varies with age and in combination with distinct pathological conditions. One of such factors influencing the neurogenesis and NSC homeostasis is the oxidative damage, which links the disturbed neurogenesis with the pathology of Alzheimer's disease.

## 3. Neurogenesis in Alzheimer's Disease

Neurogenesis is enhanced in a variety of acute neurological disorders, such as ischemia and epilepsy, but the effects of more chronic neurodegeneration are uncertain. It has been suggested that ageing is associated with decreased neurogenesis (Chen et al. 2017). However, in age-related AD, the obtained data so far are inconsistent, and different reports suggested that the neurogenesis was either enhanced or diminished. It has been recently demonstrated that post-mortem brain tissue from healthy adults aged 43–87 displays a modest decline in neurogenesis with age, while brain tissue from AD patients aged 52-97 was characterized by a sharp and progressive drop in neurogenesis (Moreno-Jimenez et al. 2019). Importantly, Moreno-Jimenez et al.'s findings suggest that the AD brain assures no appropriate environment for neurogenesis, yet in the absence of canonical hallmarks of the disease. It suggests that (co)-causal factors in the course of AD remain to be uncovered. Moreno-Jimenez et al. highlighted further need of the research on adult neurogenesis in AD as so far, existing studies are based on *postmortem* samples—remarkably rarely well preserved—or imperfect models of transgenic animals or nonhuman cell lines transfected with mutated human proteins.

The majority of the studies performed on transgenic animals expressing the mutant APP demonstrated decreased neurogenesis either in the DG or in both the DG and the SVZ (Feng et al. 2001, Haughey et al. 2002, Wen et al. 2002, Wang et al. 2004, Donovan et al. 2006, Wolf et al. 2006, Zhang et al. 2007). However, in the studies using APP-Swe-mutated transgenic mice, neurogenesis was enhanced likely due to the presence of oligomeric A*β* (Jin et al. 2004, Chevallier et al. 2005, Lopez-Toledano & Shelanski, 2007). In transgenic mice expressing mutated presenilin 1 (PS1), enhanced as well as decreased neurogenesis was observed (Chevallier et al. 2005). Increased neurogenesis was reported in the SVZ of young mice expressing mutant APP and PS1, as well as following in vivo and in vitro exposure to A*β*1–42 [18]. Conversely, neurogenesis was markedly reduced in ageing AD mice together with increased A*β* plaque formation as a result of oxidative stress [19]. Analysis of postmortem brain tissues from humans clinically diagnosed with AD revealed a reduction in NPCs in the SVZ [20], but an increase in the DG (Jin et al. 2004). Early in the disease, oligomeric A*β* may transiently promote the generation of immature neurons from NSCs (Waldou and Shetty 2008). In experiments on 3xTg-AD mice, neurogenic capabilities seemed to be impaired in both SVZ and SGZ of the hippocampal DG (Rodríguez et al. 2008, 2009). Next, several shreds of evidence link the tau protein to neurodevelopment and adult neurogenesis showing immature neurons, identified by doublecortin (DCX) and neurogenic differentiation factor (neuroD), immunoreactive to phosphorylated tau in hippocampal DG of adult rat brain and postmortem in AD brains [21]. Disturbed neurogenesis was found to be accompanied by an increase in the content of hyperphosphorylated tau protein in NSCs, which impaired neuronal integrity and functionality of the daughter neurons in the hippocampus (Hollands et al. 2017).

Furthermore, the relationship between neurogenesis and AD is well illustrated by the example of APP and PS1. PS1 regulates the metabolism of several players in neurogenesis, including Notch-1, EGF, *β*-catenin, and cAMP response element binding protein (CREB) (Bonds et al. 2015, Inestrosa and Varela-Nallar 2014). Knock-in of PS1 resulted in a significant reduction of NSCs and neuroblasts (Gadadhar et al. 2011). Interestingly, oxidative stress upregulates PS1 in NSCs, contributing to the overproduction of A*β* (Oda et al. 2010). Soluble APP, cleaved by *α*-secretase, stimulates proliferation of NSCs (Zhou et al. 2011) and increases differentiation of human NSCs (Lazarov et al. 2012, Kwak et al. 2006). Finally, the dysfunction of mutated APP in familial AD (FAD) mouse model was found to be linked with the compromised branching of dendritic tree (Sun et al. 2009; Bonds et al. 2015) and imbalanced GABAergic and glutamatergic input (Sun et al. 2009).

With regard to the above data suggesting in the majority an impairment of neurogenesis in AD, increasing a neurogenic potential seems to have a strong therapeutic impact. Indeed, an induction of neurogenesis with simultaneous enriching of the neuronal environment via increased levels of brain-derived neurotrophic factor (BDNF) mimicked the benefits of exercise on cognition, suggesting possible therapeutic use [22]. Another more direct evidence of the therapeutic utility of NSCs has been recently provided. It has been evidenced that transplanted NSCs fully integrate and restore the cognition ability in the mouse model of AD [23]. Summing up, numerous data have shown a link between AD and neurogenesis, paying attention to its importance both in the context of prevention and early treatment.

Although the majority of the aforementioned studies demonstrated a decline in a number of newborn neurons during pathological ageing in AD, the reason of that remains unclear. Also, it is little known about synaptic activity and functionality of newborn neurons in the healthy adult brain. Nonetheless, the decline in neurogenesis in AD suggests it may be an early contributor to the disease progression and, thus, proneurogenic therapeutic strategies are needed. In the light of various methodological approaches used thus far, it is important to take into consideration the common procedures for the preparation of human samples or for standardized cellular reprogramming to obtain human NSCs as a model, as this may lead to a reduction in tissue integrity, limiting the discovery of pathological contributors of AD and other neurological disorders.

## 4. NSCs and Oxidative Stress in Alzheimer's Disease Brain

NSCs are essential for the recovery of the nervous system overcoming various pathological conditions such as injuries or neurodegenerative disorders, linked with oxidative damage. NSCs are activated at sites of such loss and subsequently differentiate into neurons, astrocytes, and oligodendrocytes (Clarke et al. 2000). However, inappropriate management of the cellular redox status might contribute to accelerated CNS ageing and neuropathology. Changes in the functions of redox-sensitive proteins may be important for defining key aspects of stem cell proliferation and differentiation, neuronal maturation, and neuronal plasticity.

Under physiological conditions, ROS levels were found to be elevated in proliferating NSCs. Conversely, a reduction in the level of ROS was related to a decline in cell growth and proliferation for NPCs (Le Belle et al. 2011). Understanding a complete mechanism of oxidative regulation, with an emphasis on the precise ranges of ROS concentration, meets challenges of the future practical application of NSC-based therapy.

There are several mechanisms and pathways related to redox-sensitive oxidative signalling in NSCs. One of the ROS-dependent mechanism driving self-renewal of NSCs is fatty acid oxidation (FAO) (Xie et al. 2016). NSCs demonstrated a sustained decrease in oxygen consumption and the proliferation upon inhibition of FAO (Stoll et al. 2015). Another important redox-sensitive player in the balance between proliferation and differentiation of NSCs is the transcriptional factor FoxO3. Deficiency of the FoxO3 protein resulted in preventing the self-renewal of NSCs and impaired the ability to generate particular neural lineages. This deficiency was followed by preserving the resting activity of NSCs and preventing oxidative-sensitive differentiation (Renault et al. 2009). FoxO3 protects NSCs by increasing the expression of superoxide dismutase 2 (SOD), cyclin-dependent kinase Inhibitor 2A (CDKN2A), or ATM kinase, which target ROS for destruction (Bigarella et al. 2014). Furthermore, the redox-responsiveness of NSCs has also been attributed to the epidermal growth factor (EGF) and basic fibroblast growth factor (bFGF2). The EGF and bFGF receptor signalling, occurring through ROS-driven modifications of cysteine and methionine residues on activated tyrosine kinases and inactivated phosphatases, might affect survival and death of NSCs [4]. Moreover, the anaerobic glycolysis as an energy source for NSCs remains in contrast to oxidative phosphorylation in neurons (Zheng et al. 2015). Accordingly, induction of mitochondrial activity and oxidative phosphorylation in NSCs was followed by an increased ROS production and consequent inhibition of their proliferation and switch to cell differentiation (Prozorovski et al. 2015). As mentioned above, a critical role in neuronal development and plasticity is played by NADPH oxidases (NOXs). For instance, pharmacological inhibition of the NOX complex and the use of antioxidants significantly inhibited the proliferation of NPCs (Yoneyama et al. 2010). Moreover, NOX2 knockout mice exhibited a decrease in the number of proliferating progenitors in the adult hippocampus, suggesting that basal ROS production sustained by NOX2 is required for NPC maintenance (Dickinson et al. 2011). Most of the aspects of the contribution of NOX to proliferation/differentiation potential of NSCs/NPCs have been elegantly reviewed elsewhere (Bórquez et al. 2016). It has also recently been described that NSCs switch between proliferation and quiescence by reducing intracellular levels of ROS by prion protein, leading to an arrested growth of NSCs as well as migration and neurite outgrowth [24]. Overall, unbalanced oxidation and collateral damages of various cellular macromolecules have been recognized to contribute to pathological ageing of NSCs.

Next, the hypoxia conditions are also essential for the modulation of differentiation/proliferation balance of stem cells. Hypoxia promotes both newborn and adult neurogenesis *in vivo*, while oxygen and ROS also play a role in the *in vitro* neuronal differentiation (Douglas et al. 2016). Both hypoxia and redox-sensitive signalling by altering cell proliferation and differentiation balance may trigger neuroblastoma development or contribute to neurodegeneration by interfering with physiological neurogenesis (Vieira et al. 2011). More recently, hypoxia has been reported to stimulate proliferation by increasing HIF-1*α* expression and activating Wnt/*β*-catenin signalling [25], which links to AD. Concluding, both NSC and NPC proliferation depends on oxygen levels in hypoxic zones, which has been described in various studies.

Summarizing, ROS production and redox-sensitive signalling pathways are crucial for the proliferation and the direction of the differentiation of NSCs. Interestingly, under more reduced conditions, NSCs differentiate into neurons, while more the oxidized promote glial cells. As a result of the overproduction of ROS and the inclusion of inappropriate antioxidant programs in NSCs, various pathways associated both with ageing and neurodegeneration are induced. This demonstrates the need for research covering aspects of ageing of stem cells, in this case NSCs, against oxidative stress NSC, in the context of effective AD therapies.

## 5. NSC Aneuploidy and Disturbed Oxidative Stress in the Context of AD

Aneuploidy is present in the mature brain both in neurons and nonneuronal cortical cells. Aneuploidy might come from dynamically dividing progenitors within various ventricular zones. While the most aneuploid event in NSCs is the loss of a single chromosome, progenitors with up to 5 chromosome losses have been reported. Cumulatively, 10% of neurons in a healthy brain are estimated to be aneuploid (Garcia Martinez 2016). Chromosomal segregation defects were reported in mouse cortical progenitors, including supernumerary centrosomes, multipolar divisions, and lagging chromosomes. This demonstrated the presence of aneuploid NPCs with mitotic segregation defects under physiological conditions in the CNS (Yang 2003). Aneuploidy is a hallmark of cancer and developmental disorders and also considered a critical malignant factor in AD (Zekanowski and Wojda, 2009). It was found that experimentally induced aneuploidy in NSCs triggered a delayed stress response that impaired adult life span.

Interestingly, under stress conditions, most of the aneuploid NSCs continue to proliferate despite chromosomal instability, suggesting resistance to aneuploidy-associated stress, even though developing brain is considered the most sensitive tissue to aneuploidy [26]. Moreover, oxidative stress was found to promote aneuploidy together with the formation of neurofibrillary tangles in the neurogenic regions of the brain, which likely contributed to neurodegeneration in AD (Bushman and Chun, 2013). Altogether, the above suggests a strong influence of aneuploidy on NSC behaviour predisposing in the future to the degeneration of specific brain regions, as in AD.

## 6. DNA Damage Response in NSCs in AD

Oxidative stress is manifested by activation of DNA repair and DDR signalling with further implications to alterations in cell cycle checkpoints. Canonical DDR signalling pathways were found to be fully functional in NSCs [27]. Activation of DDR signalling, followed by changes in mitochondrial morphology, chromatin condensation, cell death kinetics, and activation of DNA damage sensors, was found to be present in human neurons (Martin and Chang 2018). DDR shares common proapoptotic signalling pathways with cell cycle reentry in post-mitotic neurons in AD (Folch et al. 2012). Accordingly, DDR and DNA repair involve activation of the ATM/ATR pathway, a well-known cell cycle regulator.

Consequently, we have demonstrated that following DDR induction fibroblasts, NSCs and neurons from AD patients displayed activation of the ATM/ATR signalling, manifested by recruitment and activation of ATM, but not ATR, and downstream effector Chk2 kinase, and ultimately mobilization of the p53 protein (Wezyk et al. 2018). Accordingly, another study showed that differentiation of NSCs *in vitro* was accompanied by an upregulation of ATM and the DNA-dependent kinase DNA-PK, sharp downregulation of ATR and Chk1, transient induction of p53, and the onset of apoptosis in a portion of cells (Carlesi et al. 2009). It suggests that some of the physiological actions of NSCs may be overrepresented on the pathology, such as AD, resulting in disturbed neurogenesis. Another report demonstrated that mutation of the *ATM* gene in NSCs, in Ataxia-telangiectasia (A-T) disorder, affected their proliferation, which occurred upon ROS stress and via activation of Akt and Erk1/2 pathways and by inhibition of p38/MAPK signalling (Kim and Wong 2009). It has been also shown that sensitivity to ionizing radiation, followed by the inclusion of DDR signalling, was significantly impaired in terminally differentiated astrocytes, while NSCs and precursors remained intact in this respect (Schneider et al. 2013). The latter is a notable example that the oxidative stress in NSCs and NPCs may find an outlet only in the daughter cells, such as astrocytes or neurons. Moreover, NSCs display activated canonical pathways of DDR after exposure to stress, but their descendants do not seem to have such functionality with automatic ATM repression leading to radioresistance. This suggests that active components of ATM signalling can make descendant neurons extremely sensitive to stress, as in AD. Repair of damaged DNA is critical for all the cells of the nervous system. However, the repair systems, such as nonhomologous end joining (NHEJ) and base-excision repair, are partially attenuated in postmitotic neurons (Sykora 2013). On the other hand, astrocytes retain the expression of NHEJ genes with DNA-PK, the critical player in DDR (rev. [28]).

Another vital player of DDR and cell cycle that has been pathologically altered in AD is the BRCA1 protein. BRCA1 is a multifunctional E3 ubiquitin ligase involved in maintaining genomic stability by activation of DNA damage-induced cell cycle checkpoint, DNA damage repair, protein ubiquitination, chromatin remodelling, and transcriptional regulation and apoptosis (Wu et al. 2010). Our group has found an increased content of phosphorylated activated BRCA1 which was at the same time recruited into the cytosol in fibroblasts, neurons, and neuroepithelial stem cells of FAD patients (Wezyk et al. 2018). Similar to our data, BRCA1 was found to be upregulated and cytosolic relocalized in *postmortem* brains derived from late-onset sporadic AD patients (Evans et al. 2007, Mano et al. 2017). Moreover, BRCA1 co-aggregated with tau protein and contributed to DNA fragmentation in both *in vitro* in APP-mutated neuroblastoma cells and *in vivo* in 3×Tg-AD mice (Mano et al. 2017). Opposing studies suggested that AD brains were characterized by a depletion of the content of total BRCA1; however, they did not test the content of the activated phosphorylated protein (Suberbielle et al. 2015). The above results indicated the increasing importance of players in DDR, such as BRCA1, in the process of neuronal death in AD. Next, BRCA1 was described as the critical player of the proliferation of neuronal progenitors. Precisely, cytosol-specific BRCA1 isoform missing exon 11 (BRCA1-*Δ*ex11) was found indispensable for apoptosis of NSCs during their asymmetrical divisions. BRCA1 acted as a centrosomal factor in establishing the cellular polarity of the neural progenitors through the ATM kinase, while its loss induced the p53-dependent proapoptotic pathway (Pao et al. 2014). This study provided essential data on the impact of altered BRCA1 expression levels in NSCs. It is in agreement with our studies showing significant upregulation of the BRCA1-*Δ*ex11 in neural precursors in AD, which was particularly well pronounced upon induction of oxidative DNA damage with doxorubicin (Wezyk et al. 2018). Upregulated BRCA1-*Δ*ex11 in neural progenitors in AD may predispose them to death or affect daughter neurons. What is more, as mentioned, BRCA1 is one of the key players of cell cycle checkpoints. In agreement, our group has suggested that the disturbed cell cycle arrest signalling in AD fibroblast and lymphocytes may occur by the BRCA1-ATM/Chk2/p53/p21 axis (Wojsiat et al. 2017, Wezyk et al. 2018). Accordingly, the p21 signalling has a role in the proliferation arrest of NSCs, both at the G1 and G2 phase (Patricio et al. 2013). Likewise, p21 controls the expansion of subependymal NSCs by limiting genetic defects upon the downregulation of the pluripotency-associated transcription factor SOX2 (Marqués-Torrejón et al., 2013). Another recent report demonstrated that overexpression of BRCA1 in NSCs promoted cell survival and functional recovery after transplantation in ischemic stroke [29]. In detail, Xu et al. suggested that BRCA1 could suppress apoptosis in NPCs and modulate oxidative stress in neurons. Interestingly, BRCA1 was upregulated by oxygen-glucose deprivation/reoxygenation. In addition, the RING finger domain and the BRCT domain of BRCA1 could physically interact with p53 in NSCs under such conditions. The crosstalk between BRCA1 RING finger domain and p53 was responsible for p53 ubiquitination and degradation. These findings are in line with the data we obtained for NSCs from patients with AD (Wezyk et al. 2018).

Besides, cell cycle checkpoint abnormalities were also found in AD neurons, which was reflected in the recruitment of the BRCA1-dependent phosphatase Cdc25C, crucial for cell cycle reentry (Wezyk et al. 2018). Interestingly, in line with the notion of DDR-driven differentiation of adult stem cells, BRCA1 together with the Fanconi anaemia complementation group D2 (FANCD2) was described to prevent the epithelial-to-mesenchymal transition in human mammary epithelial cells (Wang et al. 2016). The above data together suggest that modulation of the level and activity of BRCA1 and its network is crucial for various brain conditions, starting with physiological neurodevelopmental processes and ending with pathological ageing.

Additionally, DDR exhibits ATM-dependent activation, but not apoptosis of quiescent NSCs (qNSCs). The ATM-driven response was found to function to remove damaged progenitor cells and neuroblasts and to help in repopulation by activation of qNSCs (Barazzuol et al. 2017). Other studies testing the potential of DDR in NSCs revealed time-dependent neural differentiation in NSCs manifested by upregulation of ATM and DNA-PK, downregulation of ATR and Chk1, and induction of p53. The response to DNA lesions was characterized by the phosphorylation of ATM and some of its substrates, such as Chk2 and p53, and formation of the *γ*-H2AX nuclear foci [30]. These data are again in agreement with our data suggesting upregulation of ATM, downregulation of ATR, and induction of p53 in AD cells (Wezyk et al.2018). However, self-renewal-promoting protective culture conditions impacted negatively on the viability of NSCs following DNA damage-induced cell cycle exit (Schneider 2014). The latter suggests that particular DDR status is needed either for death or proliferation or differentiation signals in NSCs.

Summing up, the need for a better understanding of DDR in NSCs occurring against various stress factors has been recently summarized with a recommendation of several valuable protocols [31]. Altogether, the above data indicate the importance of DDR in NSC proliferation and differentiation potential, which can be severely affected by oxidative stress in AD and other neurodegenerative disorders such as telangiectasia and ataxia with depletion of ATM. Further research is needed to assess in detail the significance and potential of modulation of various components of DDR in NSCs upon oxidative stress, including new candidates such as BRCA1, recently pinpointed in the context of AD.

## 7. iPSC-Derived Neural Cells as a Potential Diagnostic Tool in AD

Most diagnostic clinical tests in the study of AD pathogenesis focus on memory defects and general cognitive dysfunction, while other symptoms remain unsolved or even overlooked. Not surprisingly, there is still a lack of precise and reliable biomarkers for early diagnosis of the disease. The current diagnosis of AD is rather a prognosis of 4 to 5 years before the onset of the symptoms, which is thus far too late for effective prevention or slowdown of the disease. The current diagnosis is based on either genetic screening or testing of biochemical markers in CSF. Genetic screening is carried out against well-known genetic variants in PSEN1, PSEN2, and APP, limited to early-onset family cases, accounting for only about 5% of all cases or less-known variants in TREM2 or APOE4 attributable to late-onset and sporadic cases. Diagnostic biochemical tests are based on the ELISA for p-tau and various amyloid peptides in CSF. The mentioned genetic and biochemical tests are preceded or carried out in parallel with standard clinical and neuropsychological tests [32]. Therefore, novel high-throughput diagnostic methods are needed to indicate disease in advance. The ability to generate human neurons and glia cells from iPSC can increase our understanding of the underlying biology of neurodegenerative disorders, including AD. Thus, it can contribute to more efficient diagnostics and then be the good starting material for high-throughput diagnostic screening. iPSC-derived neuronal cells can be a comprehensive tool for discovering novel, faster diagnostic approaches. The search for new diagnostic methods using iPSC should be based on the discoveries of new key players in AD. An example of such diagnostic approach is the screening of selected new inflammation-related parameters in combination with well-known disease-factors (i.e., MiR-106b, miR-146b, miR-181a, miR-200a, miR-34a, miR-124b, miR-153, miR-155, A*β*1-42 monomer, A*β*1-42 oligomer, UCHL1, NLRP3, Tau, STAT3, SORL1, Clusterin, APOE3, APOE4, Nogo-A, IL-13, and Visfatin) [33]. Such a set of markers associated with inflammation can be screened in peripheral blood as well as in neural cells derived from iPSC cultured in both 2D and 3D systems, providing the necessary neuronal context. Another example was provided with several hundred compounds tested using neurons derived from iPSC to identify drugs that can improve the effect of A*β*42-induced toxicity. As a consequence, several small-molecule inhibitors have been proposed that can block the toxic effect of amyloid [34]. Among the identified small inhibitors, prognostic factors for diagnosis can be further selected. This study suggests that very good insight into the disease can be obtained from screening using iPSC-derived neurons, both to get new mechanistic insights and to prepare new diagnostic approaches. Finally, chemical library screens performed on iPSC-derived neurons can provide data on new diagnostic methods and new therapeutic agents that are effective in patient cells, without translating rodent results for human use.

Importantly, for the reliability of future diagnostic methods and therapies, one critical aspect should be identified regarding the differentiation status of iPSC-derived cells used for screening, as this may result in a wide variation in results as described by others. For example, sensitivity to *γ*-secretase inhibitors [35] and immunotherapy for A*β* [36] has been shown to differ between early and late stages of differentiation of iPSC-derived cells. Another critical condition to be met for future high-throughput use of iPSC-derived neural cells for disease modeling, diagnostic testing, and drug search is well-established 3D culture conditions. In this respect, the standardization of existing 3D systems for the needs of modelling the human brain, ageing for decades, being a complex multidimensional structure, is becoming the most desirable condition.

An important example of the use of iPSC cells for translational purposes has been provided by research on the use of human iPSC to detect human pathogenic viruses using a PCR-based screening system. Importantly, these screenings used various iPSCs from skin fibroblasts, peripheral blood mononuclear cells (PBMCs), or mesenchymal stem cells generated by various reprogramming systems, both integrating and nonchromosomally integrating. The cell type of origin and the reprogramming method play an important role in process efficiency [37]. Another example of the use of iPSC to diagnose disease was a cell-based phenotypic diagnostic test for identifying long QT syndrome to identify pathogenic variants [38]. An inspirational diagnosis model composed of iPSC-derived cardiomyocytes in combination with machine learning was implemented to distinguish signals from cells from a subject carrying a disease-causing mutation or from a healthy individual [39]. To date, iPSC has been successfully used for drug screening such as anti-A*β* screening using human iPSC-derived neurons to treat Alzheimer's disease. These studies confirmed that iPSC-derived neuronal cells express functional *β*- and *γ*-secretases involved in the production of A*β* and can be used to screen for anti-A*β* drugs but warned that the use of these iPSC-neurons requires an adequate degree of neuronal differentiation and maturity [35]. The same applies to the mentioned problem of the requirements for the standardization of 3D cultures of iPSC-neuronal organoids, potential for use and for diagnostic purposes.

Overall, the usefulness of iPSCs from AD patients to study the mechanism of disease progression, with an emphasis on those at increased risk of disease, has been deftly revised by Sullivan and Young-Pearse [39]. The usefulness of iPSC to discover new pathways involved in AD pathology is increased by combining with other studies, e.g., genome-wide association screens. In this respect, iPSC-derived cells, including neuronal cells, are a comprehensive, high-throughput tool for translational genetics into biological functions.

In summary, the question arises whether neurons derived from iPSC could be a viable diagnostic tool. Generally, one can ask whether iPSC cells of different origins (fibroblasts, PBMCs, and mesenchymal cells) can be routinely collected from patients and used for a diagnostic platform. This creates a paradigm of personalized iPSC for the patient, which seems to be the only way to accurately diagnose pathologies of disease subtypes. On the other hand, the current equipment and labour costs associated with the iPSC approach mean that the direct clinical applications of this technology on a large scale are still quite dismissed in time. However, thanks to the optimization of reprogramming and differentiation protocols for iPSC derivatives, especially the 3D conditions, we can approach the more high-throughput practical application of this approach.

## 8. Challenges and Future Perspectives

NSCs are now seen as a hopeful therapeutic approach in many neurological disorders, both neurodevelopmental and neurodegenerative, including AD. However, before safe therapeutic use becomes familiar, a balance between NSC proliferation and differentiation should be ensured. One of the critical factors for this balance is adequate redox signalling, which is strictly dependent on the ROS concentration. To meet all therapeutic requirements, it is necessary to be prepared for various types of stress, including oxidative stress. That is why it is indispensable to create a good AD research model capturing all the features of human ageing and AD-specific. Such features include epigenetic signatures, telomere biology, oxidative stress markers, mitochondrial structural changes, amyloid and tau pathology, and the expression pattern of all six tau isoforms typical of mature neurons. So far, transgenic animals and cell lines transduced with human mutant proteins (APP, PS1) do not seem to meet these expectations fully. Meanwhile, cellular reprogramming technology enabled the derivation of iPSCs from somatic cells of AD patients, further transduced into NSCs and differentiated to neurons. Also, cellular reprogramming technology provided induced human neurons (iNs) converted directly from fibroblasts from patients with AD. In short, both iPSC and iN neurons have certain advantages and disadvantages that complement each other. iPSC-neurons can be burdened with rejuvenation and erased epigenetic memory but provide solid, fully neuronal cell lines. In turn, iNs maintain signatures of the origin (epigenetic ageing signatures, DNA damage stress markers, changes in mitochondrial structures, and telomere shortening, etc.) but may bear some fibroblast features.

Nevertheless, the resulting cells, derived from both iPSC and iN, seem to reproduce the pathogenesis of AD well and have been successfully used in numerous *in vitro* studies (Yagi et al. 2011, [40–42]). Besides, the advantage of iPSC is the ability to use self-organizing 3D organoids or mini brains [43–45]. The 3D organoids mimic many aspects of the human brain and can be genetically manipulated by the CRISPR/Cas9 gene editing to improve modelling of the pathophysiology of the disease. Despite clear progress, it remains a challenge to model the ageing process in the current brain organoids [46]. Up to now, this has been attempted in Parkinson's disease model by expressing progerin, a shortened form of lamin A associated with premature ageing [47], or by lowering the telomerase level [48]. Despite the challenges mentioned, it is expected that research using the best-suited models will help identify the points of elimination of the negative impact of oxidative stress on the therapeutic potential of NSC. In brief, by controlling oxidative stress, the therapeutic effectiveness of NSC can be improved. Therefore, there is an urgent need for further extensive research into the biology of NSCs and the consequences of their instability, especially in the context of new emerging players in AD, such as BRCA1 related to DNA damage and oxidative stress reaction.

In conclusion, further research is needed to explain the pathomechanisms of AD at the earliest basis, which may be related to abnormalities already in neurogenesis. Such studies could enable new therapeutic approaches by switching from standard biochemical drugs to cellular therapies. Therefore, disorders in NSCs, affecting their neurogenic potential controlled by oxidation, should be further investigated, providing a deeper insight into AD pathology and a therapeutic tool.

## Supplementary Materials

Supplementary Materials Asymmetric/symmetric divisions of neural stem cells (NSCs) are regulated by redox-sensitive signalling involving DNA damage response (DDR) pathways. This is crucial for maintaining optimal brain repair/plasticity potential in neurogenic niches. Unsustainable redox signalling may affect the activity of NSCs and thus adult neurogenesis, contributing to the onset of Alzheimer's disease.
