# Supplementary material for: Oxidative DNA Damage Signalling in Neural Stem Cells in Alzheimer's Disease
Source: Oxid Med Cell Longev. 2019 Nov 13;2019:2149812. doi: 10.1155/2019/2149812 (PMC6877938; doi:10.1155/2019/2149812)

Figure:

Asymmetric/symmetric divisions of neural stem cells (NSCs) are regulated by redox-sensitive signalling involving DNA damage response (DDR) pathways. This is crucial for maintaining optimal brain repair/plasticity potential in neurogenic niches. Unsustainable redox signalling may affect the activity of NSCs, and thus adult neurogenesis, contributing to the onset of Alzheimer's disease.

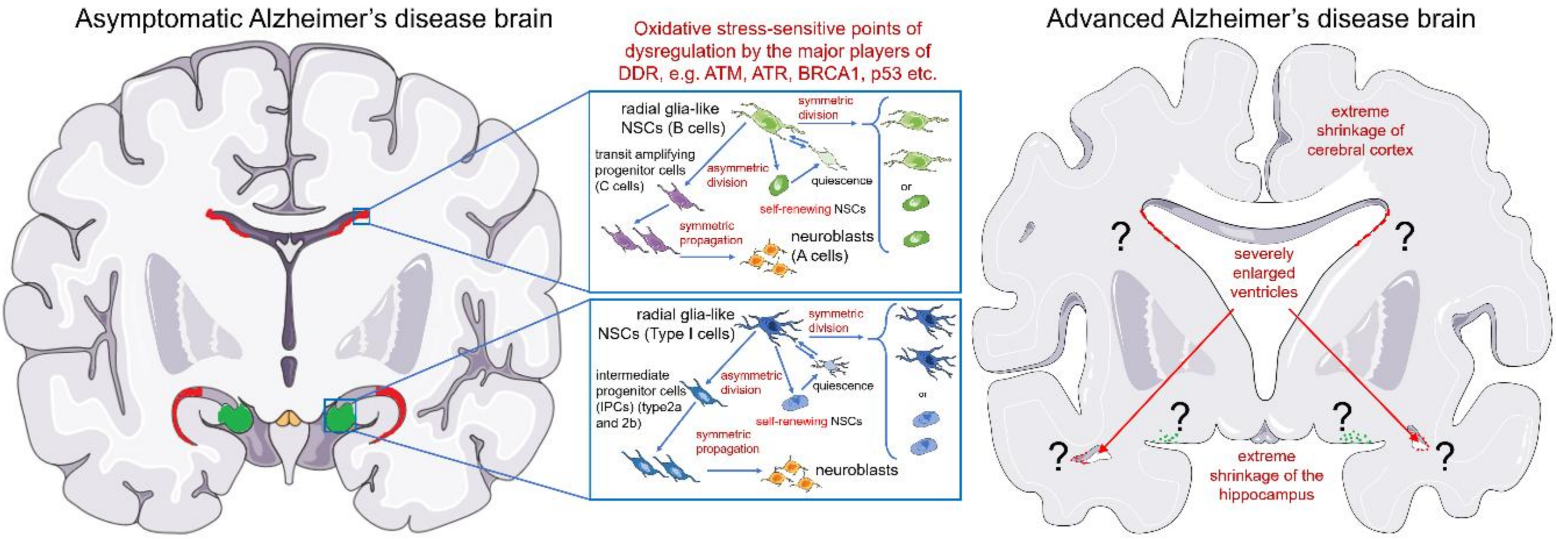

Supplement: Supplementary Materials — Asymmetric/symmetric divisions of neural stem cells (NSCs) are regulated by redox-sensitive signalling involving DNA damage response (DDR) pathways. This is crucial for maintaining optimal brain repair/plasticity potential in neurogenic niches. Unsustainable redox signalling may affect the activity of NSCs and thus adult neurogenesis, contributing to the onset of Alzheimer's disease. [file 2149812.f1.pdf]
